# Replication Efficiency of Contemporary Highly Pathogenic Avian Influenza A(H5N1) Virus Isolates in Human Nasal Epithelium Model

**DOI:** 10.3201/eid3205.260053

**Published:** 2026-05

**Authors:** Meaghan Flagg, Christopher J. Winski, Bridget G. Brackney, Tessa R. Lutterman, Johan A. Ortiz-Morales, Brandi N. Williamson, Emmie de Wit

**Affiliations:** Author affiliation: National Institute of Allergy and Infectious Diseases, National Institutes of Health, Hamilton, Montana, USA

**Keywords:** influenza, viruses, respiratory infections, zoonoses, influenza A virus, highly pathogenic avian influenza, H5N1, clade 2.3.4.4b, upper respiratory tract, virus replication, innate immune response

## Abstract

Replication of influenza A virus in human nasal epithelium affects transmissibility and disease. We compared virus replication and immune responses in human nasal epithelium infected with seasonal and highly pathogenic avian influenza A(H5N1) viruses. Contemporary H5N1 viruses replicated better than the historical isolate; however, interferon response to B3.13 genotype viruses was dampened.

Since March 2024, a total of 70 human cases of highly pathogenic avian influenza (HPAI) A(H5N1) have been reported in the United States as a result of sporadic spillover events from poultry and dairy cattle (*1*). HPAI H5N1 clade 2.3.4.4b genotype B3.13 was responsible for many of the early cases (*2*). On January 31, 2025, HPAI H5N1 clade 2.3.4.4b genotype D1.1 was detected in dairy cattle (https://www.aphis.usda.gov/news/program-update/aphis-confirms-d11-genotype-dairy-cattle-nevada-0); D1.1 was later identified in humans (*1*). Those spillover events sparked global health concerns about the potential for large-scale spread of clade 2.3.4.4b HPAI H5N1 viruses and their risk to human and animal health.

Seasonal influenza A and HPAI H5N1 viruses both cause severe respiratory disease despite different tissue tropisms. Seasonal influenza A viruses primarily infect the upper respiratory tract (URT), whereas HPAI H5N1 viruses preferentially replicate in the lower respiratory tract (LRT). This contrast in tissue affinity is explained by differences in receptor specificity and has been implicated in transmission efficiency (*3*). Specifically, the URT predominantly expresses sialic acids linked to galactose by an α-2,6 linkage; the LRT expresses sialic acid linked to galactose via α-2,3. Despite the inefficient human-to-human transmission of HPAI H5N1 viruses, recent emergence and circulation of new genotypes in mammals emphasize the need to characterize these novel viruses in relevant respiratory tract models. Here, we compare the replication kinetics and host innate immune responses in human nasal epithelium of several seasonal influenza A virus isolates and historical and contemporary HPAI H5N1 virus isolates of 3 different genotypes.

## The Study

The nasal epithelium is the primary site of entry for influenza A virus in humans. Structurally, nasal tissue is composed of goblet, ciliated, and basal cells that produce mucin and form tight junctions as host defense mechanisms (*4*). The ability to study the nasal epithelium in vitro has substantially improved with recent advances in airway model development. The Mattek EpiNasal (https://www.mattek.com) tissue model is derived from primary human nasal epithelial cells and accurately recapitulates the in vivo mucociliary phenotype. We used the model to study growth kinetics of 8 influenza A virus isolates (Table; Appendix Figure 1) and the host response to infection.

**Table:** Influenza A virus isolates used in study of replication efficiency of contemporary highly pathogenic avian influenza A(H5N1) virus isolates in human nasal epithelium model*

Isolate	Subtype	Clade	Genotype	Known mammalian adaptions	Symptoms, disease severity	References	GISAID identifier
A/Brisbane/59/2007	H1N1	NA	NA	PB2 E627K	Unknown	(*5*)	EPI_ISL_356921
A/New York/470/2004	H3N2	NA	NA	PB2 E627K	Unknown	(*6*)	EPI_ISL_8959
A/Vietnam/1203/2004	H5N1	1	NA	PB2 E627K	Severe respiratory distress resulting in fatality	(*7*)	NCBI: txid284218
A/Texas/37/2024	H5N1	2.3.4.4b	B3.13	PB2 E627K	Mild respiratory symptoms and conjunctivitis	(*2*)	EPI_ISL_19027114
A/bovine/Ohio/B24-OSU-342/2024	H5N1	2.3.4.4b	B3.13	PB2 M631L	NA	(*8*)	EPI_ISL_19178076
A/mountain lion/MT/1/2024	H5N1	2.3.4.4b	B3.6	None	Mountain lion found dead	(*9*)	EPI_ISL_19083124
A/Wyoming/01/2025	H5N1	2.3.4.4b	D1.1	PB2 E627K	Severe respiratory disease requiring hospitalization	https://www.cdc.gov/bird-flu/spotlights/ h5n1-response-02262025.html	EPI_ISL_19749443
A/Nevada/10/2025	H5N1	2.3.4.4b	D1.1	PB2 D701N	Conjunctivitis		EPI_ISL_19726293

*All virus isolates were sequenced and found to be identical to sequences deposited in GISAID (https://www.gisaid.org) or GenBank. NCBI, National Center for Biotechnology Information; NA, not available; PB, polymerase basic.

**Figure 1:**
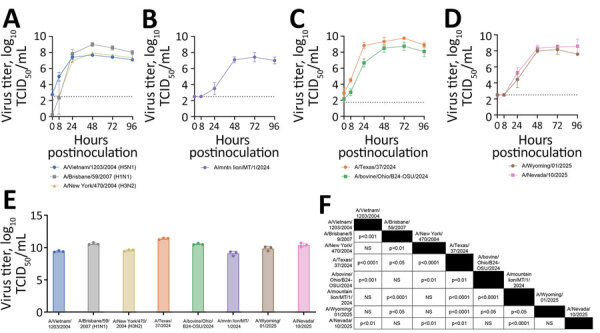
Replication of seasonal and highly pathogenic avian influenza (HPAI) A viruses in human nasal epithelium cultures in study of replication efficiency of contemporary HPAI H5N1 virus isolates in human nasal epithelium model. A–D) Virus replication in historical (A) and HPAI H5N1 genotype clade 2.3.4.4b B3.13 (B), B3.6 (C), and D1.1 (D) virus strains. We inoculated Mattek EpiNasal cultures (https://www.mattek.com) with a multiplicity of infection of 0.1 TCID_50_ per cell. We harvested apical supernatant at 0, 8, 24, 48, 72, and 96 hours postinoculation and titered on MDCK cells. Data points and error bars represent the geometric mean +SD of 3 biologic replicates from a single donor; dashed line indicates lower limit of detection. E) Area under the curve of data from 0–96 hours postinoculation as shown in panels A–D. Error bars indicate SD. F) Statistical analysis of data in panel E performed using 1-way analysis of variance with multiple comparisons (Tukey). NS, not significant; TCID_50_, 50% tissue culture infectious dose.

HPAI H5N1 isolate A/Texas/37/2024 (B3.13 genotype) replicated most efficiently in nasal tissue even when compared with seasonal isolates (Figure 1). Although we noted differences in replication kinetics between viruses of the same genotype, the B3.13 and D1.1 genotype isolates replicated more efficiently than the historical HPAI H5N1 isolate A/Vietnam/1203/2004. The B3.6 HPAI H5N1 isolate A/mountain lion/MT/1/2024 replicated least efficiently (Figure 1). Presence of known mammalian adaptations of polymerase basic (PB) 2 E627K, PB2 D701N, and PB2 M631L was associated with more efficient virus replication (Table; Figure 1).

**Figure 2:**
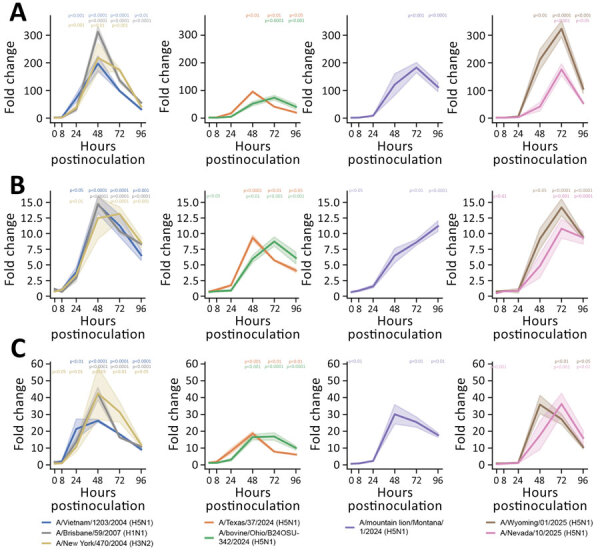
Induction of interferon-stimulated genes in human nasal epithelium infected with seasonal influenza A and highly pathogenic avian influenza (HPAI) A(H5N1) viruses in study of replication efficiency of contemporary HPAI H5N1 virus isolates in human nasal epithelium model. We inoculated Mattek EpiNasal cultures (https://www.mattek.com) with a multiplicity of infection of 0.1 50% tissue culture infectious dose per cell and extracted RNA from cells at 0, 8, 24, 48, 72, and 96 hours postinoculation. We ran quantitative reverse transcription PCR using primers (Integrated DNA Technologies, https://www.idtdna.com) to detect interferon-stimulated gene 15 (A), interferon-induced transmembrane protein 3 (B), and myxovirus resistance 1 (C) for historical and clade 2.3.4.4b highly pathogenic avian HPAI H5N1 genotype B3.13, B3.6, and D1.1 virus strains. Lines indicate median; shading indicates 95% CI. We normalized data to internal controls (ACTB and GAPDH) and calculated fold change relative to timepoint-matched mock-infected controls. Fold change is reported for 3 biologic replicates. We performed statistical analysis using 2-way analysis of variance with Dunnett posttest. p values are shown by asterisks in colors matching isolates: *p<0.05; **p<0.01; ***p<0.001; ****p<0.0001.

**Figure 3:**
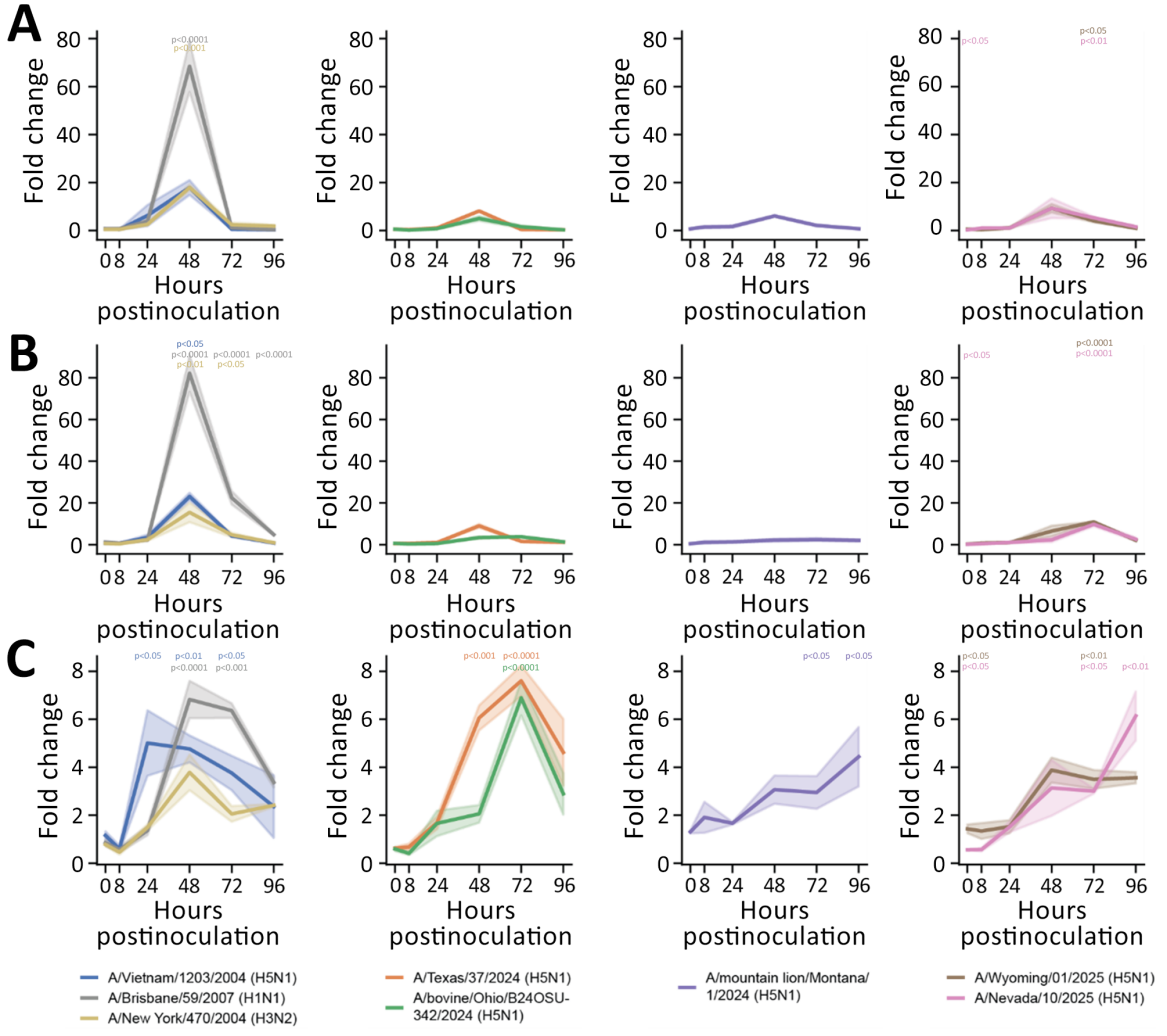
Induction of proinflammatory cytokines in human nasal epithelium infected with seasonal influenza A and highly pathogenic avian influenza (HPAI) A(H5N1) viruses in study of replication efficiency of contemporary HPAI H5N1 virus isolates in human nasal epithelium model. We inoculated Mattek EpiNasal cultures (https://www.mattek.com) with a multiplicity of infection of 0.1 50% tissue culture infectious dose per cell. RNA was extracted from cells at 0, 8, 24, 48, 72, and 96 hours postinoculation. We ran quantitative reverse transcription PCR using primers (Integrated DNA Technologies, https://www.idtdna.com) to detect interleukin 6 (A), tumor necrosis factor α (B), and interleukin 1β (C) for historical and clade 2.3.4.4b HPAI H5N1 genotype B3.13, B3.6, and D1.1 strains. Lines indicate medians; shading indicates 95% CIs. We normalized data to internal controls (ACTB and GAPDH) and calculated fold change relative to timepoint-matched mock-infected controls. Fold change is reported for 3 biologic replicates. We performed statistical analysis using 2-way analysis of variance with Dunnett posttest. p values are shown by asterisks in colors matching isolates: *p<0.05; **p<0.01; ***p<0.001; ****p<0.0001.

Next, we assessed the host innate immune response to infection by quantifying interferon-stimulated gene (ISG) 15, interferon-induced transmembrane protein 3, myxovirus resistance 1, and proinflammatory cytokines interleukin 6 (IL-6), tumor necrosis factor α, and interleukin 1β (IL-1β), as previously described (*10*). B3.13 genotype viruses induced lower ISG responses than did D1.1 and historical HPAI H5N1 viruses, as well as seasonal influenza A viruses (Figure 2). Of note, dampened ISG response occurred despite high levels of virus replication (Figure 1). In contrast, the HPAI H5N1 A/mountain lion/MT/1/2024 isolate replicated to the lowest titers yet resulted in induction of ISGs similar to A/Vietnam/1203/2004 (Figure 2). ISGs peaked earlier and higher in human nasal epithelium inoculated with D1.1 A/Wyoming/01/2025 isolated from a severe case than for D1.1 A/Nevada/10/2025 isolated from a mild case (Table; Figure 2). We observed a different pattern for induction of proinflammatory cytokines. Only infection with the seasonal influenza A viruses and A/Vietnam/1203/2004 resulted in increased expression of all 3 proinflammatory cytokines. All other isolates only induced notable IL-1β expression, but not IL-6 or tumor necrosis factor α (Figure 3).

HPAI H5N1 virus replication can be affected by the physiologic temperature of the human nasal mucosa, for which the reference is 33°C (*11*,*12*). To address the potential effect of temperature on virus replication, we quantified virus replication kinetics at 37°C and 33°C in MDCK cells. At 8 hours postinoculation, the titers of HPAI H5N1 virus isolates were lower at 33°C than at 37°C (Appendix Figure 2). However, all viruses reached the same maximum titer at 33°C and 37°C. In addition, the relative difference observed between viruses at 37°C were similar at 33°C, suggesting that adjusting the temperature in the nasal epithelial cultures to 33°C would not have substantially altered our results.

The sporadic infections of mammals with HPAI H5N1 viruses coupled with the emergence of new genotypes raises questions regarding the pandemic potential of such viruses. Our use of a human nasal epithelium model to compare virus replication kinetics of and host immune responses to seasonal influenza A viruses and HPAI H5N1 clade 2.3.4.4b viruses from different genotypes showed that HPAI H5N1 B3.6 isolate A/mountain lion/MT/1/2024 was the least efficient at replicating in the nasal epithelium. Of note, the HPAI H5N1 A/bovine/Ohio/B24-OSU-342/2024 isolate lacks the canonical PB2 E627K substitution but has the PB2 M631L substitution, which was recently shown to increase polymerase activity in mammalian cell culture and increase virulence in a mouse model (*8*).

Previously, we compared replication kinetics of contemporary versus historical HPAI H5N1 viruses in a human alveolar organoid LRT model (*10*). In the LRT, the historical HPAI H5N1 virus replicated more efficiently than the contemporary HPAI H5N1 isolates, in contrast to our observations in the URT in this study. That finding suggests that the contemporary HPAI H5N1 viruses are better adapted to replicate in the human nasal epithelium.

Our study provides novel insights regarding the biological differences among influenza A viruses in the human nasal epithelium. Specifically, we observed a correlation between mammalian adaptation and replication efficiency in the human URT. Recently published work comparing B3.13 and D1.1 isolates in human nasal epithelium observed that the D1.1 isolate replicated better than the B3.13 isolate (*13*). That study used only 1 virus per genotype, and the B3.13 isolate was not a human isolate and lacked the PB2 E627K mammalian adaptation, which likely explains the reduced virus replication. Nonetheless, despite several studies highlighting the efficient replication of contemporary HPAI H5N1 viruses in the nasal epithelium, we see no evidence of human-to-human transmission. Existing immunity could partially explain that finding. A study using ferret models found that prior exposure to seasonal influenza A(H1N1) virus significantly reduced nasal shedding of contemporary H5N1 virus and prevented infection with H5N1 in a contact transmission setting (*14*). Thus, seasonal influenza A viruses have the potential to prime the immune system and prevent infection and transmission of contemporary HPAI H5N1 viruses. Influenza A(H1N1)pdm09–like viruses are still circulating in humans, and serosurveillance of exposed dairy farm workers revealed high (66%) prevalence of pdm09 neutralizing H1N1 antibodies (*15*). Therefore, existing immunity to influenza A(H1N1)pdm09–like viruses might protect against contemporary H5N1 infection and onward transmission despite high capacity for virus replication in human nasal epithelium.

## Conclusions

Our results reveal that contemporary HPAI H5N1 isolates with known mammalian adaptations replicate more efficiently than historical HPAI H5N1 virus used. Despite high levels of virus replication, ISG induction was limited in response to B3.13 genotype virus infection. Additional studies are needed to further understand how virus replication efficiency and innate immune responses affect mammalian transmission efficiency. Existing immunity to other influenza A viruses might protect against contemporary H5N1 infection and onward transmission.

Appendix Additional information for replication efficiency of contemporary highly pathogenic avian influenza A(H5N1) virus isolates in human nasal epithelium model.
