# Supplementary material for: Replication Efficiency of Contemporary Highly Pathogenic Avian Influenza A(H5N1) Virus Isolates in Human Nasal Epithelium Model
Source: Emerg Infect Dis. 2026 May;32(5):794–9. doi: 10.3201/eid3205.260053 (PMC13175096; doi:10.3201/eid3205.260053)
Supplement: Appendix — Additional information for replication efficiency of contemporary highly pathogenic avian influenza A(H5N1) virus isolates in human nasal epithelium model. [file 26-0053-Techapp-s1.pdf]

Article DOI: https://doi.org/10.3201/eid3205.260053

EID cannot ensure accessibility for supplementary materials supplied by authors. Readers who have difficulty accessing supplementary content should contact the authors for assistance.

Replication Efficiency of Contemporary Highly Pathogenic Avian Influenza A(H5N1) Virus Isolates in Human Nasal Epithelium Model

Appendix

Methods

Viruses

The following wild-type viruses were used: H1N1 isolate A/Brisbane/59/07; H3N2 isolate A/New York/470/2004; clade 1 HPAI H5N1 isolate A/Vietnam/1203/2004; clade 2.3.4.4b, genotype B3.13 HPAI H5N1 isolate A/Texas/37/2024 (provided by Todd Davis, CDC, Atlanta, GA, USA); clade 2.3.4.4b, genotype B3.13 HPAI H5N1 isolate A/bovine/Ohio/B24OSU-432/2024 (provided by Richard Weddy, St. Jude Children's Research Hospital, Memphis, TN, USA, and Andrew Bowman, Ohio State University, Columbus Ohio, OH, USA); clade 2.3.4.4b, genotype B3.6 HPAI H5N1 isolate A/mountain lion/MT/1/2024; clade 2.3.4.4b, genotype D1.1 HPAI H5N1 isolate A/Wyoming/01/2025 (provided by CDC, Atlanta, GA, USA); and clade 2.3.4.4b, genotype D1.1 HPAI H5N1 isolate A/Nevada/10/2025 (provided by CDC, Atlanta, GA, USA). All viruses were propagated once in Madin-Darby canine kidney (MDCK) cells in infection media: minimum essential media (MEM) supplemented with 1mL L-glutamine, 50 U/mL penicillin, 50 µg/mL streptomycin, 1 x NEAA, 20 mM HEPES, and 4 µg/mL TPCK trypsin. Virus stocks were sequenced and found not to contain SNPs compared to reference sequences; amino acid differences between HPAI H5N1 isolates are highlighted in Appendix Figure 1. MDCK cells were maintained in MEM supplemented with 10% fetal bovine serum, 1 mM L-glutamine, 50 U/mL penicillin, and 50 µg/mL streptomycin, 1 x NEAA, and 20 mM HEPES.

Cells

EpiNasal transwell tissues were purchased from Mattek and maintained using the EpiNasal maintenance medium according to the manufacturer's instructions. MDCK cells were maintained in MEM supplemented with 10% fetal bovine serum, 1 mM L-glutamine, 50 U/ml penicillin, and 50 μg/ml streptomycin, 1x NEAA, 20 mM HEPES. Mycoplasma testing is performed monthly; cells remained negative throughout the study.

Growth kinetics

Mattek EpiNasal cultures (https://www.mattek.com/mattek-product/epinasal/) were inoculated with influenza viruses normalized as multiplicity of infection (MOI) in $TCID_{50}$ per cell. Briefly, cells growing on two Mattek EpiNasal transwell cultures were dissociated into a single-cell suspension and counted to calculate the average number of cells present on the transwells. EpiNasal tissues were inoculated with the indicated viruses in the apical chamber of the transwell at MOI 0.1 in a total volume of 150μl EpiNasal maintenance media for 1 hr at 37°C + 5% CO_2. The inoculum was removed and tissues were washed with PBS. The basolateral medium was removed and replaced with 5 mL of fresh EpiNasal maintenance medium. Apical supernatants were harvested at 0, 8, 24, 48, 72, and 96 hrs post-inoculation: 300 uL of EpiNasal maintenance media was added to the apical chamber of each well and incubated at room temperature for ~1 min. The 300 uL of supernatant was collected into a vial and this process was repeated with an additional 300 uL of EpiNasal maintenance media to collect a total of 600 uL of apical supernatant. After harvesting of supernatant, cells were lysed in 300 µl RLT lysis buffer at 0, 8, 24, 48, 72, and 96 hrs post-inoculation.

MDCK cells were inoculated with a MOI of 0.1 with the different virus isolate and incubated for 1 hr at 37°C or 33°C + 5% CO_2 and washed. After washing, plates were returned to 37°C or 33°C, respectively. Supernatants were harvested at 0, 8, 24, 48, and 72 hrs post-inoculation.

Virus titration

Endpoint titrations were performed on culture supernatants in MDCK cells seeded in 96-well plates. Samples were serially diluted 10-fold in infection media. Cells were washed with PBS prior to the addition of culture supernatant. Cells were incubated at 37C + 5% CO_2. After 3 days, hemagglutination assays were performed on supernatants to determine whether infectious

influenza A virus was present. The 50% tissue culture infectious dose ($TCID_{50}$) was calculated using the Spearman-Karber method.

Quantification of IFN-stimulated gene and pro-inflammatory cytokine expression

RNA was extracted from 1.2 x 10^6 cells using the QIAGEN RNeasy kit (QIAGEN) following the tissue extraction instructions. Quantitative reverse transcription PCR was performed using primers (Integrated DNA Technologies) to detect ISGs: ISG15, IFITM3, and MX1; and pro-inflammatory cytokines: IL-6, TNFα, and IL-1β. Data were normalized to internal controls (ACTB and GAPDH) and fold change was calculated relative to timepoint-matched, mock-infected controls. Fold change is reported for 3 biological replicates.

Multiple sequence alignment

Translation of protein sequences from consensus virus stock sequences was conducted using Biopython (version 1.85). Amino acid sequences were aligned using Clustal Omega (version 1.2.4). Multiple sequence alignments were visualized using pyMSAviz (version 0.5.0).

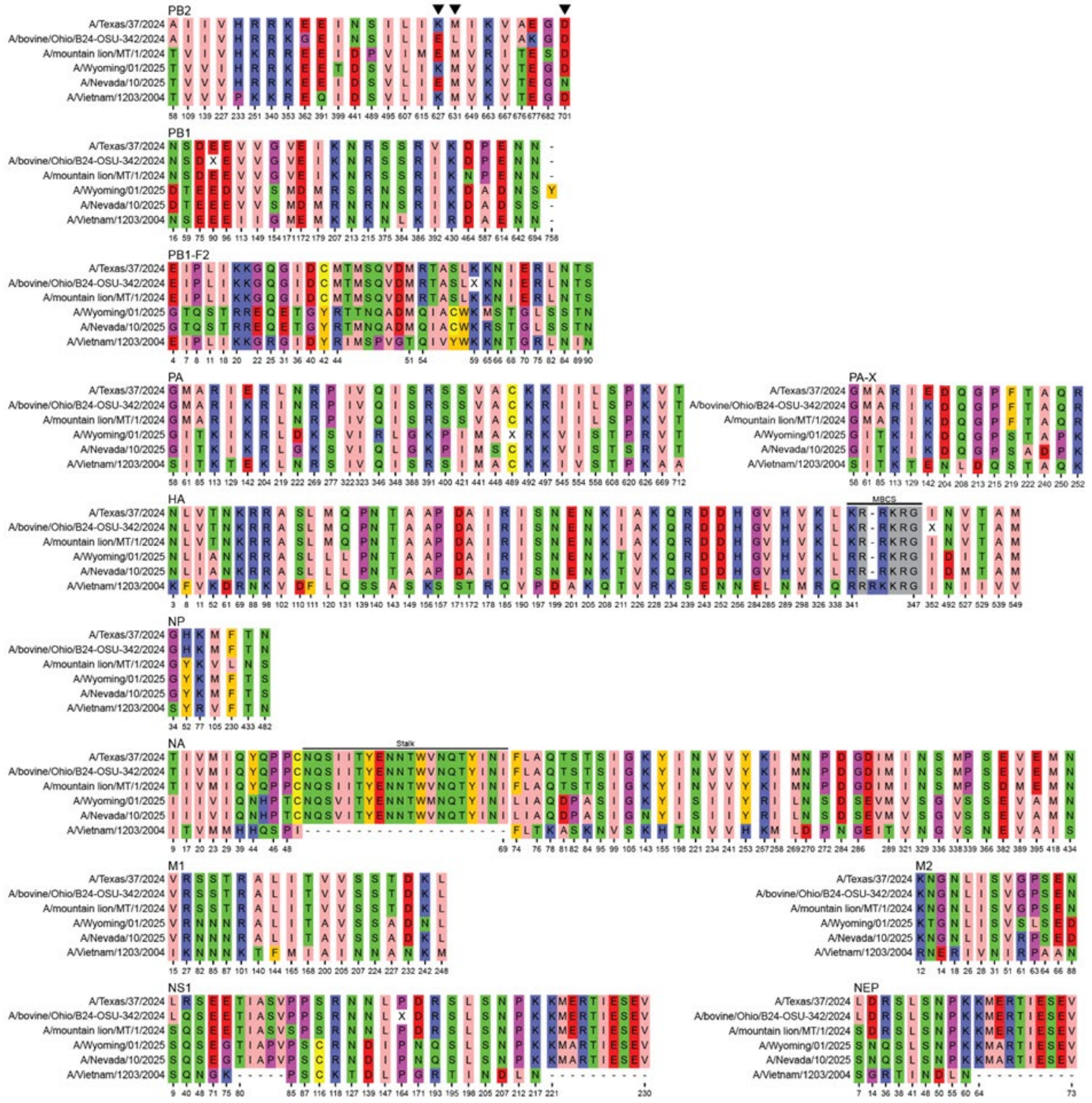

Appendix Figure 1. Amino acid differences between HPAI H5N1 viruses used in this study. Translated protein sequences derived from the consensus virus stock sequence were aligned using Clustal Omega. All positions with amino acid differences are shown. Residue numbers correspond to the beginning of the open reading frame in A/Texas/37/2024. Triangles indicate positions associated with known mammalian adaptations referred to in the Table in the main text. MBCS; multi-basic cleavage site.

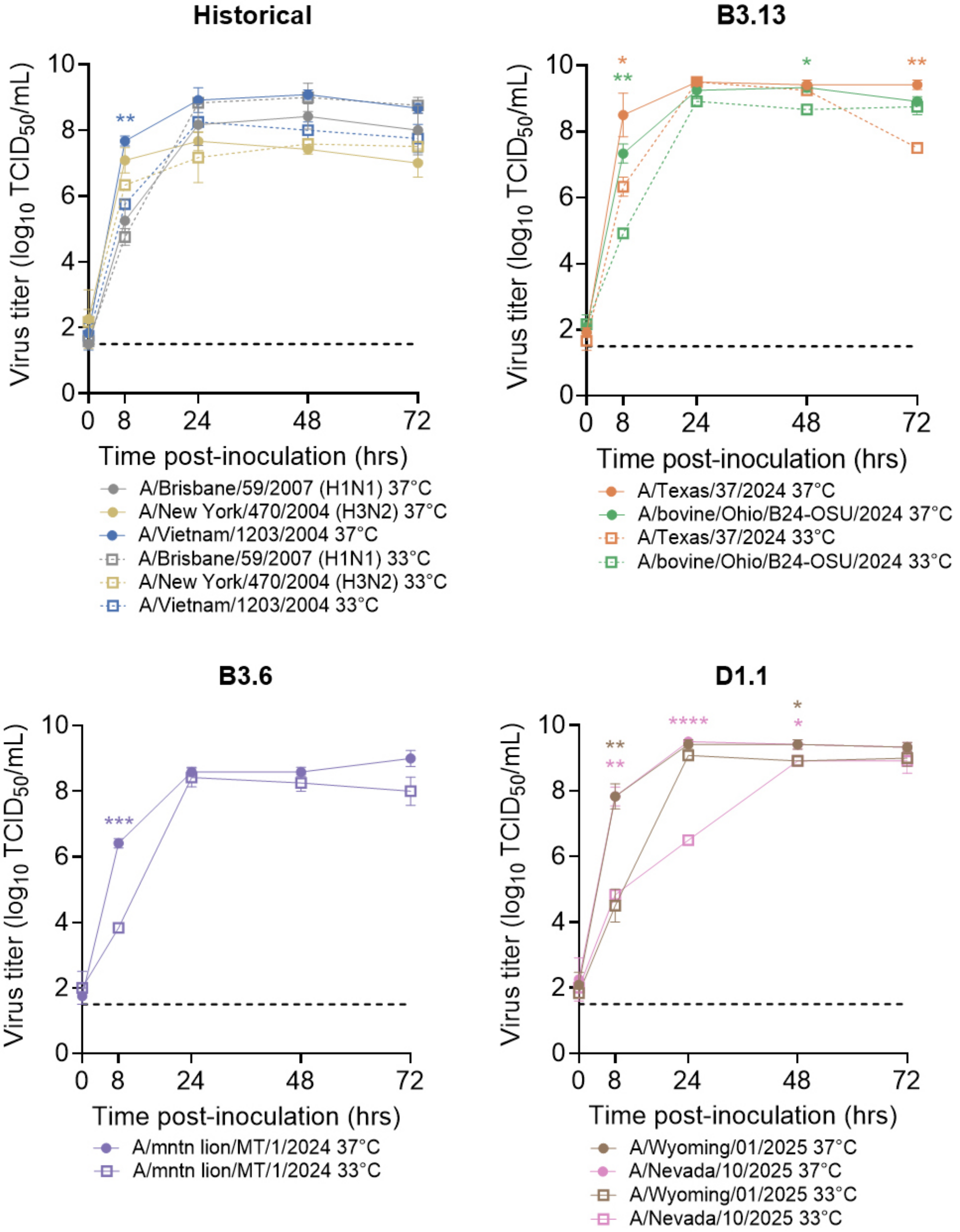

Appendix Figure 2. Comparative virus replication of seasonal and highly pathogenic avian influenza viruses at 33°C and 37°C. We compared three historical isolates, and clade 2.3.4.4b HPAI H5N1 genotype B3.13, B3.6, and D1.1 influenza A virus strains We inoculated MDCK cells with a multiplicity of infection of 0.1 $TCID_{50}$, incubated for 1 hour at 37°C or 33°C with 5% CO_2, and washed. Temperatures were maintained as during virus inoculation throughout the sampling period. We harvested supernatants at 0, 8, 24, 48, and 72 hours postinoculation and titered on MDCK cells. Data points and error bars represent the geometric mean ±SD; dashed line indicates lower limit of detection. $TCID_{50}$, 50% tissue culture infectious dose.
